# Warp analysis research pipelines: cloud-optimized workflows for biological data processing and reproducible analysis

**DOI:** 10.1093/bioinformatics/btaf494

**Published:** 2025-09-09

**Authors:** Kylee Degatano, Aseel Awdeh, Robert Sidney Cox, Wes Dingman, George Grant, Farzaneh Khajouei, Elizabeth Kiernan, Kishori Konwar, Kaylee L Mathews, Kevin Palis, Nikelle Petrillo, Geraldine Van der Auwera, Chengchen (Rex) Wang, Jessica Way

**Affiliations:** Data Sciences Platform, Broad Institute of MIT and Harvard, Cambridge, MA 02142, United States; Data Sciences Platform, Broad Institute of MIT and Harvard, Cambridge, MA 02142, United States; Data Sciences Platform, Broad Institute of MIT and Harvard, Cambridge, MA 02142, United States; Data Sciences Platform, Broad Institute of MIT and Harvard, Cambridge, MA 02142, United States; Data Sciences Platform, Broad Institute of MIT and Harvard, Cambridge, MA 02142, United States; Data Sciences Platform, Broad Institute of MIT and Harvard, Cambridge, MA 02142, United States; Data Sciences Platform, Broad Institute of MIT and Harvard, Cambridge, MA 02142, United States; Data Sciences Platform, Broad Institute of MIT and Harvard, Cambridge, MA 02142, United States; Data Sciences Platform, Broad Institute of MIT and Harvard, Cambridge, MA 02142, United States; Data Sciences Platform, Broad Institute of MIT and Harvard, Cambridge, MA 02142, United States; Data Sciences Platform, Broad Institute of MIT and Harvard, Cambridge, MA 02142, United States; Data Sciences Platform, Broad Institute of MIT and Harvard, Cambridge, MA 02142, United States; Data Sciences Platform, Broad Institute of MIT and Harvard, Cambridge, MA 02142, United States; Data Sciences Platform, Broad Institute of MIT and Harvard, Cambridge, MA 02142, United States

## Abstract

**Summary:**

In the era of large data, the cloud is increasingly used as a computing environment, necessitating the development of cloud-compatible pipelines that can provide uniform analysis across disparate biological datasets. The Warp Analysis Research Pipelines (WARP) repository is a GitHub repository of open-source, cloud-optimized workflows for biological data processing that are semantically versioned, tested, and documented. A companion repository, WARP-Tools, hosts Docker containers and custom tools used in WARP workflows.

**Availability and implementation:**

The WARP and WARP-Tools repositories and code are freely available at https://github.com/broadinstitute/WARP and https://github.com/broadinstitute/WARP-tools, respectively. The pipelines are available for download from the WARP repository, can be exported from Dockstore, and can be imported to a bioinformatics platform such as Terra.

## 1 Introduction

Efficient sequencing technology produces petabytes of data every day (Papageorgiou *et al.* 2018). To effectively combine disparate datasets and turn them into meaningful insights, researchers need analysis tools and data that adhere to Findable, Accessible, Interoperable, and Reusable (FAIR) practices (Wilkinson *et al.* 2016, Khan *et al.* 2019). Cloud computing allows for efficient resource sharing and scalability of computing resources (Ewels *et al.* 2020, Schatz *et al.* 2022). Multiple cloud-based platforms like Terra and Seven Bridges are available to ease the researcher cloud transition. Large scientific consortia are increasingly turning to these platforms for reproducible analyses. This migration to cloud computing means researchers require new pipelines that are optimized to harness the expanse of cloud resources. While the number of cloud-optimized pipelines is growing, many are developed to meet niche research needs and may lack the robust systems for documentation, versioning, and testing which are crucial to FAIR.

**Figure 1.**
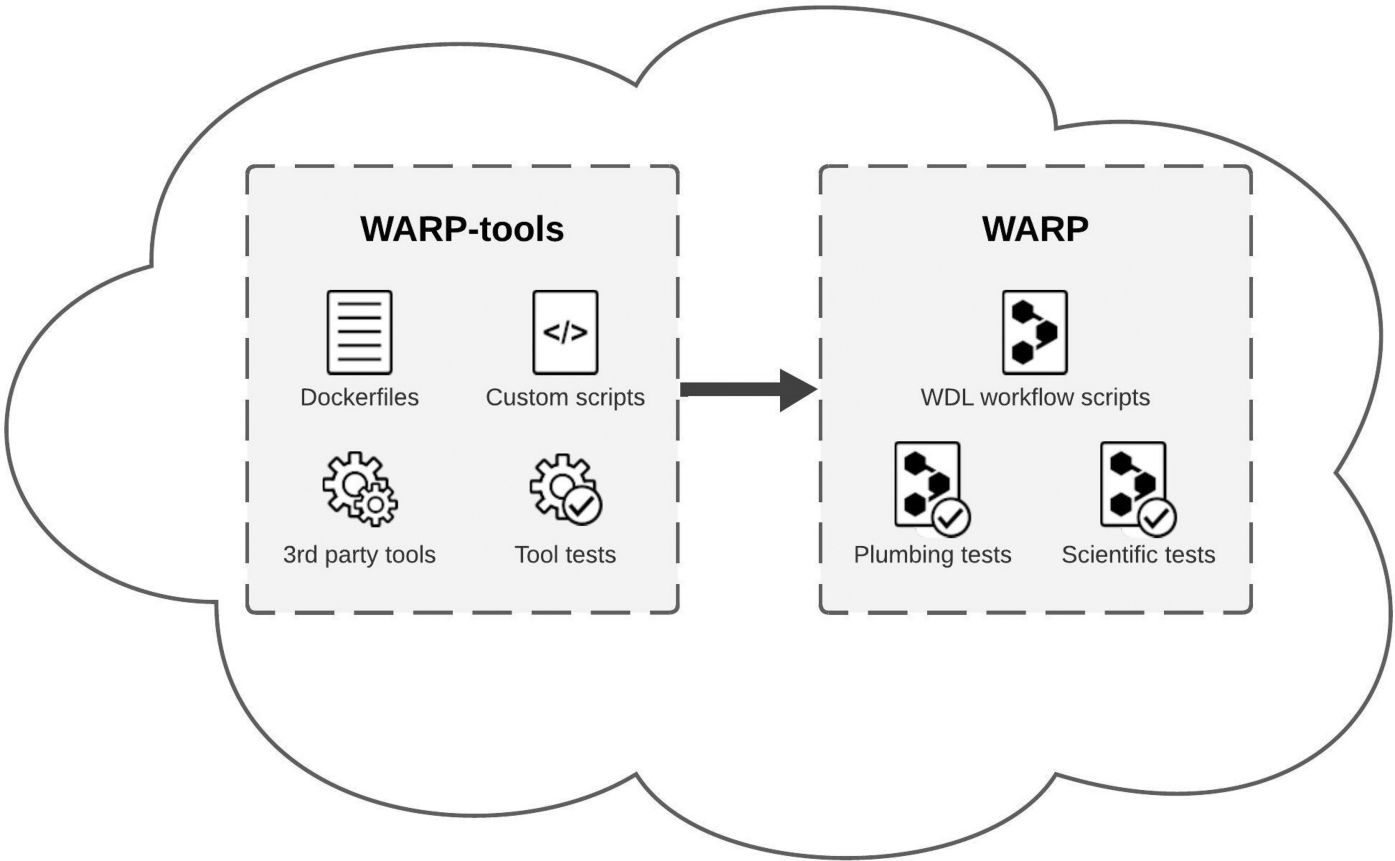
An illustrated overview of the WARP and WARP-tools repositories. The WARP repository hosts a collection of cloud-based workflow scripts, or pipelines (Table 1, available as supplementary data at *Bioinformatics* online), for processing high-throughput sequencing data along with plumbing and scientific tests used to validate pipeline releases. The WARP-tools repository offers a suite of Dockerfiles, custom scripts, third-party tools, and tool tests essential for data preprocessing and analysis performed within WARP pipelines. Together, these repositories provide a collection of cloud-optimized pipelines for genomic data processing that are accessible and FAIR.

The Warp Analysis Research Pipelines (WARP) repository (Degatano *et al.* 2021) in GitHub is a collection of scalable, cloud-optimized pipelines designed for processing a broad range of “omic” datasets (Fig. 1; Table 1, available as supplementary data at *Bioinformatics* online). Initially developed in the Workflow Description Language (WDL) to meet the requirements of researchers using Terra as their cloud analysis platform, WARP has transformed into a repository of modular, Docker-based code that is developed, tested, and scientifically vetted in collaboration with community scientists and global consortia. The repository has an open-access infrastructure under a BSD-3 license that includes all workflow code, and reference files, as well as a companion repository (WARP-tools) containing Dockerfiles and custom tools used in WARP pipelines (Fig. 1). Each pipeline is semantically versioned, documented, publicly released in WARP and Dockstore (O’Connor *et al.* 2017). They are available to run in local environments or cloud bioinformatics platforms such as Terra (https://app.terra.bio), or be modified for additional compatible environments (https://broadinstitute.github.io/warp/).

## 2 Implementation

WARP is designed to provide the research community with accessible, portable, and rigorously tested pipelines for analyzing diverse omic datasets. Pipelines are accessible to the entire research community from the WARP releases page, Dockstore, and Terra. When a pipeline is released, it is first tagged with its version number and packaged on the WARP releases page, where it can be discovered using Wreleaser. Each release download includes the workflow, its subtasks, and example configuration files that can be used to run the pipeline. After release, each tagged pipeline is automatically pushed to Dockstore, where it can be downloaded, run, or exported to cloud-based analysis platforms like Terra. The WARP team maintains a dedicated Terra workspace for each pipeline; the workspaces are preloaded with the pipeline workflow, example data, and instructions for running the workflow (Table 1, available as supplementary data at *Bioinformatics* online).

WARP pipelines can be deployed either locally or in the cloud. They are written in WDL, which can be deployed by a portable execution engine like Cromwell. Workflows deploy software from public Docker images, that can be pulled from cloud repositories like Azure Container Registry and Google Container Registry. The majority of the pipelines have testing data hosted in public Google bucket storage that researchers can use to try the pipelines. WARP’s use of public cloud resources ensures pipelines are portable and executable on a wide variety of computing platforms.

WARP implements healthy software development practices by semantically versioning workflows. This system provides researchers with insight into how different code changes may affect outputs and necessitate data reprocessing. A patch change represents engineering updates, such as memory changes and variable name changes, that do not affect scientific outputs. A minor change reflects changes to outputs within a level of reasonable noise, such that the adjustment does not fundamentally affect the scientific outputs and does not necessitate any data reprocessing. A major change alters outputs in a way that may require reprocessing. Together, this system reliably captures the state of the data, inputs and outputs, informing the community how pipeline changes impact data processing and downstream analysis.

The WARP infrastructure enables rigorous pipeline testing, ensuring that all updates to workflow code function, produce accurate and consistent scientific outputs, and are appropriately reviewed. To this end, the repository contains three branches: a develop branch for initial pipeline changes and testing, a staging branch that collects many pipeline commits for release, and a master branch that has been scientifically tested and validated on full-size example data. All pipeline contributions start with a pull request (PR) to the develop branch. Merging from develop to staging and from staging to release requires a mandatory review from a minimum of two reviewers. Changes to scientific outputs may require an additional review from a dedicated scientific collaborator. This system ensures the reliability and scientific accuracy of each released pipeline.

In addition to human review, WARP deploys four types of automated testing to remove overhead from the pipeline developer: syntax, versioning, engineering, and scientific. Syntax tests use WOMtool to lint. Versioning tests identify WDL or Docker code impacted by PR changes, verify that changelogs are updated, and prompt documentation for scientific output changes. Engineering, or “plumbing,” tests use small, customized example data to effectively test all pipeline components, including optional pipeline tasks. A PR which passes the syntax and versioning tests automatically launches the engineering tests for updated pipelines. Scientific tests use full-size example data to confirm that pipeline changes produce outputs that exactly match the scientific reference datasets. Multiple reference datasets are chosen to catch special data edge cases, like high contamination or low coverage. WARP uses call caching, which keeps the results of successful tests and reuses them whenever there is no pipeline change. This means that the scientific tests will only run one time successfully for each release, and similarly that the engineering tests will only run one time successfully for each time they are changed in either develop or staging (tests which pass in develop do not need to be rerun in staging). Overall, WARP combines versioning, human review and automated testing to ensure that each pipelines’ components function harmoniously and reliably for the scientific community.

## 3 Results and discussion

WARP exemplifies how collaborative efforts across consortia and individual researchers can develop robust, scalable pipelines that drive advances in large-scale data processing. The WARP repository contains open-source, cloud-optimized workflows that reproducibly and scalably maximize the use of community data through portable workflow management systems, Docker containers, and comprehensive documentation (Wilkinson *et al.* 2016).

Driven by community collaborations, WARP pipelines have successfully processed data from multiple scientific partners, including the Broad Genomics Platform, Human Cell Atlas (HCA) and the BRAIN Initiative (Ament *et al.* 2023, Hawrylycz *et al.* 2023). For example, the Whole Genome Analysis (WGS) pipeline is the GATK Best Practices pipeline used for the Broad Genomics Platform and greater scientific community (Van der Auwera *et al.* 2013). It is one of the most frequently launched WARP pipelines in Terra, with over 300 workflow submissions in 2024. It is actively used by the community to benchmark and refine algorithmic performance (Gürsoy *et al.* 2020). The accessibility of the pipeline in Terra has even empowered genomics discovery by citizen scientists. These findings demonstrate that repositories developed with FAIR principles like WARP make science more accessible to the entire research community.

Other examples of WARP’s success are its single-cell RNA-seq pipelines initially developed for the Human Cell Atlas (HCA) in 2017. Multiple groups have used these single-cell pipelines for benchmarking (You *et al.* 2021) and integration into downstream tools (Li *et al.* 2020). Because of the pipelines’ modularity, other consortium such as LungMAP (Gaddis *et al.* 2024) and BICAN have modified and adapted the pipelines to meet their needs for reproducible analysis. So far, BICAN has applied six WARP pipelines to process approximately 62 million single cells across ten animal species. Notably, the Multiome workflow was independently adopted by West *et al.* (2025) to replicate BICAN single-cell processing, reinforcing the reproducibility and utility of WARP workflows beyond the original consortium. WARP has scaled cloud deployment for large consortia, while empowering citizen scientists with production-grade research tools.

WARP pipelines are accessible and have been downloaded >4500 times (GREV). In the last four years, the WARP documentation page has increased from 1400 active users in 2021 to over 12 000 active users in 2025 widely distributed across six continents. Anyone with a GitHub account can make suggestions to the existing pipeline code, or collaborate with WARP to add a new pipeline, by following WARP contribution guidelines. To add new pipelines or modify existing pipelines, we provide directions for forking WARP and deploying pipelines on a variety of execution environments. The WARP releases and documentation pages allow the community to access and apply these tools.

While WARP has made notable strides, a few usability limitations remain. Workflow language choice significantly impacts a pipeline’s portability, maintainability, and adoption. Currently, all WARP pipelines utilize WDL 1.0 due to their development within consortia projects originally intended for the Terra platform. WDL’s user-friendly syntax and support in Cromwell facilitate execution across various environments, including local, high performance computing (HPC) and cloud. However, the flexibility of Cromwell and the maturity of WDL tooling can add complexity to deployment and container integration.

The WARP repository continuously evolves to address the changing needs of the scientific community; future work may therefore include incorporating Nextflow pipelines into WARP. Nextflow has emerged as a widely adopted alternative, with growing momentum driven by the nf-core community, which hosts over 1500 modules. It uses the Java virtual machine for execution, simplifying backend setup relative to Cromwell, and provides features for optional typing and file path handling. Supporting a broader spectrum of workflow languages will increase the accessibility, interoperability, and long-term sustainability of the WARP ecosystem.

WARP pipelines are a suite of scalable and flexible workflows designed to streamline data processing in a diversity of cloud environments. While currently optimized for Google Cloud, their modular design enables easy adaptation for cross-platform flexibility. The underlying Docker images in WARP-tools are also fully portable and can be used independently of WDL, supporting execution in various workflow frameworks such as Nextflow, Snakemake, and Common Workflow Language. Moreover, WARP workflows have been successfully adapted for various platforms, including the WGS workflow for HPC environments (Powers *et al.* 2022) and the Multiome workflow for Amazon Web Services (West *et al.* 2025). Multiple pipelines have built-in cloud provider arguments, which have also enabled successful pipeline deployment in Azure. Moving forward, the WARP team plans to expand these parameters to support pipeline use across multi-cloud environments. This modularity allows the pipelines to be deployed beyond the Terra platform.

Overall, the WARP repository provides a model for cloud-optimized pipeline best practices that can be reused by the community (Fig. 1). The WARP team continues to enhance and refine pipelines, ensuring they remain accessible and cutting-edge. This commitment positions WARP as an essential resource for driving innovation in genomics and beyond.

## Supplementary Material

btaf494_Supplementary_Data

## Data Availability

The data underlying this article are available in the WARP GitHub repository, at https://github.com/broadinstitute/warp.

## References

[btaf494-B1] Ament SA , Adkins RS, Carter R et al The neuroscience multi-omic archive: a brain initiative resource for single-cell transcriptomic and epigenomic data from the mammalian brain. Nucleic Acids Res 2023;51:D1075–85. 36318260 10.1093/nar/gkac962 PMC9825473

[btaf494-B2] Degatano K, Grant G, Khajouei F et al Introducing warp: a collection of cloud-optimized workflows for biological data processing and reproducible analysis [version 1; not peer reviewed]. *F1000Research* 2021;**10**:705.

[btaf494-B3] Ewels PA , Peltzer A, Fillinger S et al The nf-core framework for community-curated bioinformatics pipelines. Nat Biotechnol 2020;38:276–8. 32055031 10.1038/s41587-020-0439-x

[btaf494-B4] Gaddis N , Fortriede J, Guo M et al LungMAP portal ecosystem: systems-level exploration of the lung. Am J Respir Cell Mol Biol 2024;70:129–39. 36413377 10.1165/rcmb.2022-0165OC PMC10848697

[btaf494-B5] Gürsoy G , Emani P, Brannon CM et al Data sanitization to reduce private information leakage from functional genomics. Cell 2020;183:905–17.e16. 33186529 10.1016/j.cell.2020.09.036 PMC7672785

[btaf494-B6] Hawrylycz M , Martone ME, Ascoli GA et al A guide to the brain initiative cell census network data ecosystem. PLoS Biol 2023;21:e3002133. 37390046 10.1371/journal.pbio.3002133 PMC10313015

[btaf494-B7] Khan FZ , Soiland-Reyes S, Sinnott RO et al Sharing interoperable workflow provenance: a review of best practices and their practical application in cwlprov. Gigascience 2019;8:giz095. 31675414 10.1093/gigascience/giz095 PMC6824458

[btaf494-B8] Li B , Gould J, Yang Y et al Cumulus provides cloud-based data analysis for large-scale single-cell and single-nucleus RNA-seq. Nat Methods 2020;17:793–8. 32719530 10.1038/s41592-020-0905-x PMC7437817

[btaf494-B9] O’Connor BD, Yuen D, Chung V et al The dockstore: enabling modular, community-focused sharing of docker-based genomics tools and workflows. F1000Research. 2017;**6**:52. 10.12688/f1000research.10137.1 PMC5333608 28344774

[btaf494-B10] Papageorgiou L , Eleni P, Raftopoulou S et al Genomic big data hitting the storage bottleneck. EMBnet J 2018;24:e910. PMC5958914 29782620

[btaf494-B11] Powers ME , Mannthey K, Sebastian P et al Deploying genomics workflows on high performance computing (HPC) platforms: storage, memory, and compute considerations. bioRxiv, 2022, preprint: not peer reviewed.

[btaf494-B12] Schatz MC , Philippakis AA, Afgan E et al Inverting the model of genomics data sharing with the NHGRI genomic data science analysis, visualization, and informatics lab-space. Cell Genom 2022;2:100085. 35199087 10.1016/j.xgen.2021.100085 PMC8863334

[btaf494-B13] Van der Auwera GA , Carneiro MO, Hartl C et al From FastQ data to high confidence variant calls: the genome analysis toolkit best practices pipeline. Curr Protoc Bioinform 2013;43:11.10.1–11.10.33. 10.1002/0471250953.bi1110s43 PMC4243306 25431634

[btaf494-B14] West NR, Arachchilage KH, Knaack S *et al*. Single-nucleus analysis reveals oxidative stress in Down syndrome basal forebrain neurons at birth. *Alzheimer's Dement* 2025;**21**:e70445. 10.1002/alz.70445 PMC12265022 40667939

[btaf494-B15] Wilkinson MD , Dumontier M, Aalbersberg IJJ et al The fair guiding principles for scientific data management and stewardship. Sci Data 2016;3:160018–9. 26978244 10.1038/sdata.2016.18 PMC4792175

[btaf494-B16] You Y , Tian L, Su S et al Benchmarking UMI-based single-cell RNA-seq preprocessing workflows. Genome Biol 2021;22:339. 34906205 10.1186/s13059-021-02552-3 PMC8672463

