# Supplementary material for: Warp analysis research pipelines: cloud-optimized workflows for biological data processing and reproducible analysis
Source: Bioinformatics. 2025 Sep 9;41(10):btaf494. doi: 10.1093/bioinformatics/btaf494 (PMC12490826; doi:10.1093/bioinformatics/btaf494)
Supplement: btaf494_Supplementary_Data [file btaf494_supplementary_data.pdf]

Supplementary Information for:

Warp Analysis Research Pipelines: Cloud-optimized workflows for biological data processing and reproducible analysis

Kylee Degatano1, Aseel Awdeh, Robert Sidney Cox III, Wes Dingman, George Grant, Farzaneh Khajouei, Elizabeth Kiernan, Kishori Konwar, Kaylee L. Mathews, Kevin Palis, Nikelle Petrillo, Geraldine Van der Auwera, Chengchen (Rex) Wang and Jessica Way

Supplementary Table 1: List of select WARP workflows

Pipeline	WARP WDL Code	Input Data	Overview	Terra Workspace	Publication (if applicable)
Exome Germline Single Sample	Exome Germline Single Sample	Human exome sequencing data	Exome Germline Single Sample Overview	Exome Germline Single Sample	Van der Auwera & O'Connor, 2020
Imputation	Imputation	Multi-sample variants calls from genotyping array data	Imputation Overview	Imputation	
Optimus	Optimus	10x Genomics V2 and V3 3' single-cell and single-nucleus data	Optimus Overview	Optimus	
Single-Cell ATAC (ATAC)	ATAC	Single-cell ATAC-seq data from nuclear isolates	ATAC Overview	Multiome with ATAC	Fang et al. (2021)

Slide-seq	Slide-seq	Spatial transcriptomic data	Slide-seq Overview	Slide-seq	
Smart-seq2 Single Nucleus Multi-Sample	Smart-seq2 Single Nucleus Multi-Sample	Single-cell data generated with Smart-seq2 assays	Smart-seq2 Single Nucleus Multi-Sample Overview	Smart-seq2 Single Nucleus Multi-Sample	
Whole Genome Germline Single Sample	Whole Genome Germline Single Sample	Human whole-genome paired-end sequencing data	Whole Genome Germline Single Sample Overview	Whole Genome Germline Single Sample	Van der Auwera & O'Connor, 2020
